# Moving Beyond “Food Deserts”: Reorienting United States Policies to Reduce Disparities in Diet Quality

**DOI:** 10.1371/journal.pmed.1001914

**Published:** 2015-12-08

**Authors:** Jason P. Block, S. V. Subramanian

**Affiliations:** 1 Obesity Prevention Program, Department of Population Medicine, Harvard Medical School, Harvard Pilgrim Health Care Institute, Boston, Massachusetts, United States of America; 2 Harvard Center for Population and Development Studies, Harvard T H Chain School of Public Health, Cambridge, Massachusetts, United States of America; 3 Department of Social and Behavioral Sciences, Harvard T H Chan School of Public Health Boston, Massachusetts, United States of America

## Abstract

Jason Block and S. V. Subramanian explore avenues for improving the health of Americans through reducing dietary inequalities and look at whether concern over "food deserts" has been taken too far.

**Summary Points**

- Most Americans fail to meet recommendations for diet quality, with disparities evident by race/ethnicity, socioeconomic status, and use of nutrition assistance programs.
- The evidence supporting the elimination of food deserts as a strategy to reduce disparities in diet quality is weak.
- More use of evidence-based policies to improve diet quality and reduce disparities in the United States is needed, specifically for school- and child care-based interventions, useful population educational strategies, changes to nutrition assistance programs, and taxation of unhealthy foods such as sugar-sweetened beverages.

## Introduction

Scientific interest in dietary patterns and the relationship between diet and disease has persisted for centuries [1]. During much of the 19th century, concerns regarding diet centered on the high prevalence of malnutrition, with a clear income gradient evident. Those living in abject poverty, often in urban centers that grew rapidly because of industrialization, were most affected. With increasing recognition of vitamin deficiencies and government-mandated vitamin fortification of foods such as bread, prevalence of “undernutrition” dramatically fell [1]. By the end of the 20th century, in industrialized countries, interest in the link between diet and disease was rekindled, except that the concern was “overnutrition,” reflected in equally dramatic increases in the prevalence of overweight and obesity [1]. The Scientific Report of the 2015 US Dietary Guidelines Committee confirms that we still don’t eat a healthful diet [2]. Food insecurity remains a problem for low-income families, but the greatest challenge is poor dietary quality. In the US, nearly all people fail to consume recommended amounts of whole grains; 80% and 90% do not eat enough fruits and vegetables, respectively [2]. In contrast, 70% of the US population eats more saturated fat and refined grains than recommended, and 90% consumes too much added sugar [2].

Poor diet quality is strongly patterned by socioeconomic status (SES). In a recent US study examining trends in dietary quality from 1999 to 2010, lower-income individuals consumed lower-quality foods, as measured by the Alternate Healthy Eating Index 2010 score, than higher-income individuals [3]. Notably, income disparities in dietary quality widened over this period, with some improvements for higher-income individuals alone. These disparities are also evident when comparing participants in nutrition assistance programs to nonparticipants. In a study of low-income individuals from 1999 to 2008, Supplemental Nutrition Assistance Program (SNAP, “food stamp”) participants had lower dietary quality and consumed fewer whole grains and more red meat, potatoes, and fruit juice than nonparticipants [4]. Among women, SNAP participants consumed more sugar-sweetened beverages than nonparticipants [4,5].

In this essay, we discuss the specific role of physical access to food and the extent to which eliminating food deserts can improve dietary quality and decrease economic and racial/ethnic disparities in dietary quality. While a variety of factors contribute to income disparities in dietary quality, considerable attention has been focused on poor physical access to healthy foods leading to “food deserts.” The White House Task Force on Childhood Obesity explicitly considered the existence of “food deserts” (see Box 1 for a formal definition) as a contributor to poor nutrition in the US and advocated their elimination by 2017 [6]. The World Health Organization has advocated similar approaches [7]. Meanwhile, a small fraction (23.5 million, approximately 7% of the population) of Americans live in food deserts; even fewer live in food deserts without access to a privately owned car (2.3 million) [8]. Yet, there appears to be governmental enthusiasm for developing programs and regulations to eliminate food deserts. For instance, the US Departments of Agriculture, Health and Human Services and Treasury joined together to develop the Healthy Food Financing Initiative (HFFI) to “improve access to healthy, affordable foods” in food deserts [9], committing US$50 million in federal money for HFFI, with other substantial federal support through tax credits and community economic development grants to fund healthy food options in food deserts. Pennsylvania, Louisiana, and New York undertook large-scale programs to bring healthy food to low-income communities, with US$30 million in public funds in Pennsylvania alone, matched by nearly US$120 million in private funds [10]. These governmental programs provided funding to catalyze public–private partnerships leading to the construction of supermarkets and grocery stores in food deserts.

**Box 1. What Is a Food Desert?**

The US Department of Agriculture considers a census tract to be a food desert if it is low income (poverty rate greater than or equal to 20% or median family income at 80% or lower of the area median family income) and at least one-third of tract residents live more than 1 mile away (or 10 miles away in the case of rural areas) from a supermarket or large grocery store [11].

## The Food Environment and Diet: What’s the Evidence?

Food deserts refer to low-income geographic areas that lack access to a supermarket or large grocery store. Eliminating food deserts could improve diet either by providing access to healthy foods for individuals seeking those foods but with prior poor access or by generating demand within communities that had limited exposure to healthy foods. In contrast, living near fast-food restaurants might lead to poor diet quality because of enhanced access to the foods sold there; restricting access to these locations might improve diet by making this food less available. It has been shown that while area poverty was not an independent predictor of access to fast food restaurants, predominantly black neighborhoods had closer access to fast food restaurants [12]. Meanwhile, evidence from longitudinal studies has raised substantial doubt about the connection between food access and health [13–16]. In these studies, no consistent relationship emerged between the food environment and body weight, for adults or children. Even studies commonly cited as providing evidence for an association between the food environment and body weight or unhealthy food consumption have found only small associations that may not demonstrate a notable public health or clinical impact [17–19]. For example, in a study using birth certificates from over 3 million pregnant women, living within 0.5 miles of a chain fast-food restaurant was associated with 50 grams of extra gestational weight gain compared to women without this exposure [18].

Four quasi-experimental studies have found similar results. One study examined the effect of a new supermarket in a Philadelphia food desert [20]. After opening, just over half of the residents of the neighborhood with the new supermarket used the supermarket for shopping; about a quarter of the residents used it as their primary source for food shopping. When compared to residents of a control neighborhood that was also a food desert, the new supermarket was associated with a perception of improved food access among residents in that neighborhood, but it was not associated with any improvements in fruit/vegetable intake or changes in BMI. A study in New York City examined the availability and intake of healthy foods before and after a new supermarket was built in a food desert [21]. Only 13% of participants in the neighborhood with the new supermarket reported shopping there, and its opening was not associated with an improvement in the availability or intake of healthy foods compared to residents of a control neighborhood. A study of a new supermarket in a Pittsburgh food desert found more positive results [22]. Residents of the neighborhood with a new supermarket had notable reductions in calories in added sugars after the opening of the supermarket, compared to the control neighborhood; there were no changes in BMI or whole grain, fruit, and vegetable intake. However, among the two-thirds of residents in the intervention community who shopped in the new store regularly, there were no improvements, beyond perceptions of healthy food access, compared to residents who rarely if ever used the new store. Investigating the flip side to food deserts, a quasi-experimental evaluation of the 2008 ban on new fast-food restaurants in South Los Angeles found no effect of the ban on fast-food consumption or obesity rates when compared to other areas in Los Angeles county [23].

Even if proximity to healthy food establishments were beneficial to health, can “healthy” food establishments be easily defined? While supermarkets typically devote more shelf space to fruits and vegetables than other food stores, unhealthy snack foods still dominate their shelves. One study in southern Louisiana and Los Angeles found that supermarkets had one-half to three-quarters more unhealthy snacks than fruit and vegetables in their stores; convenience stores and drug stores had 90% more of these unhealthy snacks than fruits and vegetables [24]. Supermarkets in low-income neighborhoods may offer even more unhealthy food [25]. Supermarkets also focus their advertising on unhealthy foods and strategically position them near the front of stores, at the ends of aisles, or at cash registers [26,27]. This practice occurs even in the most highly acclaimed “healthy” supermarkets. In addition, supermarkets may offer less healthy canned and processed foods than some smaller food stores commonly considered to be “unhealthy,” such as convenience stores and “dollar” stores [28].

People may not shop within their own neighborhoods even when a supermarket is present [29–31]. In one study in France, only 30% of participants shopped for food at the supermarket closest to their home [31]. Even if low-income people have access to a supermarket, they may still buy unhealthy foods in greater proportion than people with higher income [32]. Factors beyond proximity, especially price, may be more important when choosing a food shopping location, and there is no evidence that simply increasing access to supermarkets enhances demand for healthier items.

With unhealthy dietary consumption so prevalent, initiatives that focus on a narrow, at-risk population such as those living in food deserts without cars (less than 1% of the US population) are likely to be less effective than alternate interventions that help families make better decisions regardless of where they live and which food establishments are near their homes. Rather than assuming that “if we build it, they will come” [33] and eat healthfully, why not focus solely on policies that have more face validity, especially those that directly target economic and racial/ethnic disparities in diet quality?

## Toward Potential Solutions to Improve Diet among Low-Income Populations

Research on effective population-level strategies to improve diet remains limited. More evidence is needed to determine which interventions are best for specific populations, including low-income and minority populations that bear a greater burden of diet-related diseases. Here we discuss several feasible strategies that have the potential to lower disparities in diet quality more than eliminating food deserts.

### Improvements in Child Care and School Nutrition Programs

Interventions in child care centers across both the US and Australia have demonstrated beneficial effects on diet, physical activity, screen time, and even body weight [34–37]. For example, one Australian community-based intervention (nearly 2,500 intervention participants and over 30,000 control participants) with a strong child care center component was associated with a reduction in prevalence of overweight and obesity in the intervention community of 2.5% and 3.4% for 2-year-old children and 3.5-year-old children, respectively, compared to a 0.7% reduction for both age groups in control communities [36]. Children in the intervention communities also reduced their consumption of packaged snacks and fruit juice, and the day care centers there reported less unhealthy foods available on-site after the intervention [35]. However, studies on the effect of child care center interventions are mixed, with some studies, especially in Hispanic and black children, showing no effect [38,39].

School nutrition programs also might have some effect on the diets of children [7]. Because low-income children get more food from these programs than other children, a beneficial effect of improved nutrition standards should help lower disparities in dietary quality [40]. The US Department of Agriculture recently updated school nutrition standards, requiring the provision of healthier items in schools. These changes were required by the Healthy, Hunger-Free Kids Act of 2010. In four urban, low-income Massachusetts school districts, a recent study documented a 23% increase in selection of fruit and a 16% increase in consumption of vegetables after implementation of the new regulations [41].

### Population Educational Initiatives

The provision of easily accessible information at the point of sale could help consumers better understand the health consequences of their purchases in restaurants and food stores. Calorie menu labeling in chain food establishments, which will be implemented across the US in 2016, is a start, though evidence of effectiveness is rather limited at this point [42,43]. Revisions of the Nutrition Facts label are forthcoming and will display calories and added sugar more prominently. Front-of-package labeling for food products may be implemented in the near future Yet, a recent study in Seattle provides evidence that we should be cautious about the potential for labeling to worsen disparities. In that study, adults reported using calorie information much more often to help guide their food purchases at restaurants following the introduction of a local calorie labeling law. However, disparities in use were present by income; those making more than US$75,000 per year were nearly twice as likely to report using calorie information compared to those making less than US$35,000 [44]. Thus, while further education is needed, greater attention should be focused on how to do so without worsening disparities.

### Changes in Food Assistance Programs

Incentivizing healthy food purchases, or disincentivizing unhealthy purchases, in food assistance programs could decrease disparities in diet quality. In 2009, the Special Supplemental Nutrition Program for Women, Infants, and Children (WIC) made changes to encourage purchases of healthier foods, including adding more fruits and vegetables to the food packages the program financed. This change led retailers to stock more healthy foods [45] and was associated with a decline in juice purchases (24%) and whole milk (50%) and an increase in fresh fruits and vegetables (29% and 18%) and whole grains (3-fold increase in 100% whole grain bread, 5-fold increase in brown rice) [46–49]. SNAP, commonly referred to as the food stamp program, could do the same if the purchase of healthy foods were incentivized by increasing the value of vouchers for those families who buy more healthy foods. The Healthy Incentive Pilot, sponsored by the US Department of Agriculture, tested this concept in a study in western Massachusetts [50]. The study included all SNAP participants in one county, with 7,500 households randomized to receive US$0.30 cash back for every US$1 spent on fruits and vegetables; the other 47,595 families in the county received traditional benefits. After one year, those receiving the incentive reported consuming one-fourth of a cup more per day of fruits and vegetables and spent about US$1.19 more per month on fruits and vegetables than the control group. These higher expenditures were determined based on purchasing data from supermarkets and superstores participating in the SNAP program. Self-reported expenditures for fruits and vegetables showed a larger effect with US$78.1 versus US$72.02 spent, respectively, for intervention versus control families. Organizations such as Wholesome Wave, which operates in 31 states and the District of Columbia and caters to approximately 40,000 families, have taken this concept further, providing dollar-for-dollar matches when customers use their SNAP benefits to purchase fruit and vegetables at farmer’s markets [51]. Reviews of studies examining the effect of lower prices (or subsidies) for fruit and vegetables have found a generally positive effect on consumption, with some evidence of lower weight after the introduction of subsidies [52,53].

The benefit of healthy food subsidies is generally modest and may be amplified in the SNAP population if SNAP vouchers could not be used for unhealthy foods such as sugar-sweetened beverages. Simulation studies have found that banning the use of SNAP dollars for sugar-sweetened beverages could be cost effective (savings of nearly US$3,000 per quality-adjusted life year saved) and could modestly reduce rates of diabetes, cardiovascular disease, and obesity with an expected 15% decline in calories from sugar-sweetened beverages [54,55]. These simulations also found that fruit and vegetable subsidies in SNAP could lead to reductions in cardiovascular death, but not obesity, and may lead to greater consumption of fruits and vegetables, similar to what was found in the Healthy Incentive Pilot.

As with investigations of changes to the WIC [5,46–49], supermarkets are a natural partner for gathering evidence regarding dietary interventions and policies, especially for participants in food assistance programs. Most people, including low-income individuals and families, use supermarkets as a major source of food shopping [29,30]. Many of the larger supermarket chains offer loyalty cards that have been leveraged in prior investigations of policy changes. In the era of “Big Data,” in which large-scale data sources are being used for population health, clinical epidemiology, genetics, and surveillance initiatives [7,56], supermarkets could be encouraged to make their “Big Data” available for use in a responsible and helpful fashion, such as for policy evaluation and targeted interventions. Clinical institutions also might partner with supermarkets to better engage their patients in health education at the point when they are making dietary choices [57].

### Taxing Unhealthy Food

Taxing unhealthy foods such as sugar-sweetened beverages (SSBs) may have important benefits for dietary quality [52,53]. Based on simulation models using supermarket scanner data in the US, these taxes would likely decrease obesity rates of lower-income populations more because of higher consumption in this population. These models predict that overall overweight and obesity rates would decline modestly from taxes, with reductions in daily calorie intake of 24 to 34 calories [58–60]. These taxes would be economically regressive, costing low-income individuals more, but this additional cost may be offset by the greater health benefit on this population. Data on the outcomes of these policies are not always consistent. In a United Kingdom modeling study, SSB taxes demonstrated regressivity but no greater decline in overweight and obesity rates among low-income individuals [61]. While acknowledging the potential economic challenges, the World Health Organization has endorsed the use of fiscal policies to improve dietary quality [62]. Advertising restrictions on unhealthy foods, especially for kids, may work in concert with fiscal policies.

## Conclusion

Addressing disparities in dietary quality may have important payoffs for the health of the population: we should promote policies and programs to support these changes while studying their effectiveness. These strategies do not preclude the elimination of food deserts but rather build a necessary infrastructure to promote healthy food consumption, in any neighborhood. Many reasons, such as economic and social justice, exist to support such initiatives and to remedy the lack of healthy food availability in low-income communities. We just should not expect the reduction of food deserts to have much impact on the prevailing health crisis of our time. We need to focus our efforts on initiatives more likely to improve dietary quality and decrease disparities.

## Abbreviations

HFFI: Healthy Food Financing Initiative
SES: socioeconomic status
SNAP: Supplemental Nutrition Assistance Program
SSB: sugar-sweetened beverage
WIC: Special Supplemental Nutrition Program for Women, Infants, and Children

## References

[1] Semba RD. Nutrition and development: a historical perspective In: Semba RD, Bloem MW, editors. Nutrition and health in developing countries. 2nd edition ed. Totawa, NJ: Humana Press; 2008 p. 1–33.

[2] Scientific Report of the 2015 US Dietary Guidelines Advisory Committee: Scientific Report to the Secretary of the Department of Health and Human Services and the Secretary of Agriculture February 2015 [cited 2015 April 10]. http://www.health.gov/dietaryguidelines/2015-scientific-report/PDFs/Scientific-Report-of-the-2015-Dietary-Guidelines-Advisory-Committee.pdf.

[3] Wang DD, Leung CW, Li Y, Ding EL, Chiuve SE, Hu FB, et al Trends in dietary quality among adults in the United States, 1999 through 2010. JAMA Intern Med. 2014;174(10):1587–95. 10.1001/jamainternmed.2014.3422 25179639 PMC5924699

[4] Leung CW, Ding EL, Catalano PJ, Villamor E, Rimm EB, Willett WC. Dietary intake and dietary quality of low-income adults in the Supplemental Nutrition Assistance Program. Am J Clin Nutr. 2012;96(5):977–88. 10.3945/ajcn.112.040014 23034960 PMC3471209

[5] Andreyeva T, Luedicke J, Henderson KE, Tripp AS. Grocery store beverage choices by participants in federal food assistance and nutrition programs. Am J Prev Med. 2012;43(4):411–8. 22992359 10.1016/j.amepre.2012.06.015

[6] White House Task Force on Childhood Obesity. Solving the problem of childhood obesity within a generation Washington, D.C.: Executive Office of the President of the United States, 5 2010. 10.1089/bfm.2010.9980 20942695

[7] Beck AH. Open access to large scale datasets is needed to translate knowledge of cancer heterogeneity into better patient outcomes. PLoS Med. 2015;12(2):e1001794 10.1371/journal.pmed.1001794 25710538 PMC4339838

[8] Ver Ploeg M, Breneman V, Farrigan T, Hamrick K, Hopkins D, Kaufman P, et al Access to affordable and nutritious food—measuring and understanding food deserts and their consequences: report to congress Washington, DC: Economic Research Service, United States Department of Agriculture, 6 2009. Report No.

[9] US Department of Health and Human Services. Administration for Children & Families. Community Economic Development Projects (HHS-2012-ACF-OCS-EE-0274). [cited 2012 May 30]. http://www.acf.hhs.gov/grants/open/foa/view/HHS-2012-ACF-OCS-EE-0274.

[10] Karpyn A, Manon M, Treuhaft S, Giang T, Harries C, McCoubrey K. Policy solutions to the 'grocery gap'. Health Aff. 2010;29(3):473–80. 10.1377/hlthaff.2009.0740 20194989

[11] United States Department of Agriculture. Food deserts. [cited 2012 May 29]. http://apps.ams.usda.gov/fooddeserts/foodDeserts.aspx.

[12] James P, Arcaya MC, Parker DM, Tucker-Seeley RD, Subramanian SV. Do minority and poor neighborhoods have higher access to fast-food restaurants in the United States? Health Place. 2014;29:10–7. 10.1016/j.healthplace.2014.04.011 24945103 PMC4783380

[13] Block JP, Christakis NA, O’Malley AJ, Subramanian SV. Proximity to Food Establishments and Body Mass Index in the Framingham Heart Study Offspring Cohort over 30 Years. Am J Epidemiol. 2011;174(10):1108–14. 10.1093/aje/kwr244 21965186 PMC3208142

[14] Lee H. The role of local food availability in explaining obesity risk among young school-aged children. Soc Sci Med. 2012;74:1193–203. 10.1016/j.socscimed.2011.12.036 22381683

[15] Eid J, Overman HG, Puga D, Turner MA. Fat city: questioning the relationship between urbal sprawl and obesity. J Urban Econ. 2008;63(2):385–404.

[16] Caspi CE, Sorensen G, Subramanian SV, Kawachi I. The local food environment and diet: a systematic review. Health Place. 2012;18(5):1172–87. 10.1016/j.healthplace.2012.05.006 22717379 PMC3684395

[17] Boone-Heinonen J, Gordon-Larsen P, Kiefe CI, Shikany JM, Lewis CE, Popkin BM. Fast food restaurants and food stores: longitudinal associations with diet in young and middle-aged adults: The Cardia Study. Arch Intern Med. 2011;171(13):1162–70. 10.1001/archinternmed.2011.283 21747011 PMC3178268

[18] Currie J, Della Vigna S, Moretti E, Pathania V. The effect of fast food restaurants on obesity and weight gain. American Economic Journal: Economic Policy. 2010;2(3):32–63.

[19] Gibson DM. The neighborhood food environment and adult weight status: estimates from longitudinal data. Am J Public Health. 101(1):71–8. 10.2105/AJPH.2009.187567 21088263 PMC3000723

[20] Cummins S, Flint E, Matthews SA. New neighborhood grocery store increased awareness of food access but did not alter dietary habits or obesity. Health Aff (Millwood). 2014;33(2):283–91. 24493772 10.1377/hlthaff.2013.0512 PMC4201352

[21] Elbel B, Moran A, Dixon LB, Kiszko K, Cantor J, Abrams C, et al Assessment of a government-subsidized supermarket in a high-need area on household food availability and children's dietary intakes. Public Health Nutr. 2015:1–10 10.1017/S1368980015000282 PMC10271373 25714993

[22] Dubowitz T, Ghosh-Dastidar M, Cohen DA, Beckman R, Steiner ED, Hunter GP, et al Diet And Perceptions Change With Supermarket Introduction In A Food Desert, But Not Because Of Supermarket Use. Health Aff (Millwood). 2015;34(11):1858–68. 26526243 10.1377/hlthaff.2015.0667 PMC4977027

[23] Sturm R, Hattori A. Diet and obesity in Los Angeles County 2007–2012: Is there a measurable effect of the 2008 "Fast-Food Ban"? Soc Sci Med. 2015;133:205–11. 10.1016/j.socscimed.2015.03.004 25779774 PMC4410074

[24] Farley TA, Rice J, Bodor JN, Cohen DA, Bluthenthal RN, Rose D. Measuring the food environment: shelf space of fruits, vegetables, and snack foods in stores. J Urban Health. 2009;86(5):672–82. 10.1007/s11524-009-9390-3 19603271 PMC2729874

[25] Cameron AJ, Thornton LE, McNaughton SA, Crawford D. Variation in supermarket exposure to energy-dense snack foods by socio-economic position. Public Health Nutr.1–8. 10.1017/S1368980012002649 PMC10271308 22613746

[26] Mehta K, Phillips C, Ward P, Coveney J, Handsley E, Carter P. Marketing foods to children through product packaging: prolific, unhealthy and misleading. Public Health Nutr.1–8. 10.1017/S1368980012001231 22608304

[27] Thornton LE, Cameron AJ, McNaughton SA, Worsley A, Crawford DA. The availability of snack food displays that may trigger impulse purchases in Melbourne supermarkets. BMC Public Health. 12(1):194. 22420759 10.1186/1471-2458-12-194 PMC3386861

[28] Bustillos B, Sharkey JR, Anding J, McIntosh A. Availability of more healthful food alternatives in traditional, convenience, and nontraditional types of food stores in two rural Texas counties. J Am Diet Assoc. 2009;109(5):883–9. 10.1016/j.jada.2009.02.011 19394475

[29] Drewnowski A, Aggarwal A, Hurvitz PM, Monsivais P, Moudon AV. Obesity and supermarket access: proximity or price? Am J Public Health. 2012;102(8):e74–80. 10.2105/AJPH.2012.300660 22698052 PMC3464835

[30] Dubowitz T, Zenk SN, Ghosh-Dastidar B, Cohen DA, Beckman R, Hunter G, et al Healthy food access for urban food desert residents: examination of the food environment, food purchasing practices, diet and BMI. Public Health Nutr. 2015;18(12):2220–30. 10.1017/S1368980014002742 25475559 PMC4457716

[31] Chaix B, Bean K, Daniel M, Zenk SN, Kestens Y, Charreire H, et al Associations of supermarket characteristics with weight status and body fat: a multilevel analysis of individuals within supermarkets (RECORD study). PLoS ONE. 7(4):e32908 10.1371/journal.pone.0032908 22496738 PMC3319546

[32] Vinkeles Melchers NV, Gomez M, Colagiuri R. Do socio-economic factors influence supermarket content and shoppers' purchases? Health Promot J Austr. 2009;20(3):241–6. 19951246 10.1071/he09241

[33] Field of Dreams (1989) 1989 [cited 2015 September 15]. http://www.imdb.com/title/tt0097351/.

[34] Benjamin Neelon SE, Taveras EM, Ostbye T, Gillman MW. Preventing obesity in infants and toddlers in child care: results from a pilot randomized controlled trial. Matern Child Health J. 2014;18(5):1246–57. 10.1007/s10995-013-1359-x 24065371 PMC3965661

[35] de Silva-Sanigorski A, Elea D, Bell C, Kremer P, Carpenter L, Nichols M, et al Obesity prevention in the family day care setting: impact of the Romp & Chomp intervention on opportunities for children's physical activity and healthy eating. Child Care Health Dev. 2011;37(3):385–93. 10.1111/j.1365-2214.2010.01205.x 21276039

[36] de Silva-Sanigorski AM, Bell AC, Kremer P, Nichols M, Crellin M, Smith M, et al Reducing obesity in early childhood: results from Romp & Chomp, an Australian community-wide intervention program. Am J Clin Nutr. 2010;91(4):831–40. 10.3945/ajcn.2009.28826 20147472

[37] Fitzgibbon ML, Stolley MR, Schiffer L, Van Horn L, KauferChristoffel K, Dyer A. Two-year follow-up results for Hip-Hop to Health Jr.: a randomized controlled trial for overweight prevention in preschool minority children. J Pediatr. 2005;146(5):618–25. 15870664 10.1016/j.jpeds.2004.12.019

[38] Fitzgibbon ML, Stolley MR, Schiffer L, Van Horn L, KauferChristoffel K, Dyer A. Hip-Hop to Health Jr. for Latino preschool children. Obesity (Silver Spring). 2006;14(9):1616–25. 17030973 10.1038/oby.2006.186

[39] Fitzgibbon ML, Stolley MR, Schiffer LA, Braunschweig CL, Gomez SL, Van Horn L, et al Hip-Hop to Health Jr. Obesity Prevention Effectiveness Trial: postintervention results. Obesity (Silver Spring). 2006;19(5):994–1003. 10.1038/oby.2010.314 PMC3775499 21193852

[40] Potamites E, Gordon A. Children's food security and intakes from school meals: final report. Princeton, NJ: Mathematica Policy Research, 2010 Contract No.: Contractor and Cooperator Report No. 61.

[41] Cohen JF, Richardson S, Parker E, Catalano PJ, Rimm EB. Impact of the new U.S. Department of Agriculture school meal standards on food selection, consumption, and waste. Am J Prev Med. 2014;46(4):388–94. 10.1016/j.amepre.2013.11.013 24650841 PMC3994463

[42] Long MW, Tobias DK, Cradock AL, Batchelder H, Gortmaker SL. Systematic review and meta-analysis of the impact of restaurant menu calorie labeling. Am J Public Health. 2015;105(5):e11–24. 10.2105/AJPH.2015.302570 25790388 PMC4386504

[43] Kiszko KM, Martinez OD, Abrams C, Elbel B. The influence of calorie labeling on food orders and consumption: a review of the literature. J Community Health. 2014;39(6):1248–69. 10.1007/s10900-014-9876-0 24760208 PMC4209007

[44] Chen R, Smyser M, Chan N, Ta M, Saelens BE, Krieger J. Changes in awareness and use of calorie information after mandatory menu labeling in restaurants in King County, Washington. Am J Public Health. 2015;105(3):546–53. 10.2105/AJPH.2014.302262 25602868 PMC4330858

[45] Andreyeva T, Middleton AE, Long MW, Luedicke J, Schwartz MB. Food retailer practices, attitudes and beliefs about the supply of healthy foods. Public Health Nutr. 14(6):1024–31. 10.1017/S1368980011000061 21324231

[46] Andreyeva T, Luedicke J. Federal food package revisions: effects on purchases of whole-grain products. Am J Prev Med. 2013;45(4):422–9. 10.1016/j.amepre.2013.05.009 24050418

[47] Andreyeva T, Luedicke J. Incentivizing fruit and vegetable purchases among participants in the Special Supplemental Nutrition Program for Women, Infants, and Children. Public Health Nutr. 2014:1–9. 24809502 10.1017/S1368980014000512 PMC10270964

[48] Andreyeva T, Luedicke J, Henderson KE, Schwartz MB. The positive effects of the revised milk and cheese allowances in the special supplemental nutrition program for women, infants, and children. J Acad Nutr Diet. 2014;114(4):622–30. 10.1016/j.jand.2013.08.018 24210878

[49] Andreyeva T, Luedicke J, Tripp AS, Henderson KE. Effects of reduced juice allowances in food packages for the women, infants, and children program. Pediatrics. 2013;131(5):919–27. 10.1542/peds.2012-3471 23629613 PMC4074658

[50] Bartlett S, Klerman J, Olsho L, Logan C, Blocklin M, Beauregard M, et al Evaluation of the Healthy Incentives Pilot (HIP): final report U.S. Department of Agriculture, Food and Nutrition Service, 9 2014.

[51] Gillings D, Makuc D, Siegel E. Analysis of interrupted time series mortality trends: an example to evaluate regionalized perinatal care. Am J Public Health. 1981;71(1):38–46. 7258429 10.2105/ajph.71.1.38 PMC1619708

[52] Powell LM, Chriqui JF, Khan T, Wada R, Chaloupka FJ. Assessing the potential effectiveness of food and beverage taxes and subsidies for improving public health: a systematic review of prices, demand and body weight outcomes. Obes Rev. 2013;14(2):110–28. 10.1111/obr.12002 23174017 PMC3556391

[53] Thow AM, Jan S, Leeder S, Swinburn B. The effect of fiscal policy on diet, obesity and chronic disease: a systematic review. Bull World Health Organ. 2010;88(8):109–614. 10.2471/BLT.09.070987 PMC2908970 20680126

[54] Basu S, Seligman H, Bhattacharya J. Nutritional policy changes in the supplemental nutrition assistance program: a microsimulation and cost-effectiveness analysis. Med Decis Making. 2013;33(7):937–48. 10.1177/0272989X13493971 23811757

[55] Basu S, Seligman HK, Gardner C, Bhattacharya J. Ending SNAP subsidies for sugar-sweetened beverages could reduce obesity and type 2 diabetes. Health Aff (Millwood). 2014;33(6):1032–9. 24889953 10.1377/hlthaff.2013.1246 PMC7421782

[56] Hay SI, George DB, Moyes CL, Brownstein JS. Big data opportunities for global infectious disease surveillance. PLoS Med. 2013;10(4):e1001413 10.1371/journal.pmed.1001413 23565065 PMC3614504

[57] Lewis KH, Roblin DW, Leo M, Block JP. The personal shopper—a pilot randomized trial of grocery store-based dietary advice. Clin Obes. 2015;5(3):154–61. 10.1111/cob.12095 25873139 PMC4676909

[58] Finkelstein EA, Zhen C, Bilger M, Nonnemaker J, Farooqui AM, Todd JE. Implications of a sugar-sweetened beverage (SSB) tax when substitutions to non-beverage items are considered. J Health Econ. 2013;32(1):219–39. 10.1016/j.jhealeco.2012.10.005 23202266

[59] Lin BH, Smith TA, Lee JY, Hall KD. Measuring weight outcomes for obesity intervention strategies: the case of a sugar-sweetened beverage tax. Econ Hum Biol. 2011;9(4):329–41. 10.1016/j.ehb.2011.08.007 21940223

[60] Zhen C, Finkelstein EA, Nonnemaker J, Karns S, Todd JE. Predicting the Effects of Sugar-Sweetened Beverage Taxes on Food and Beverage Demand in a Large Demand System. Am J Agric Econ. 2014;96(1):1–25. 24839299 10.1093/ajae/aat049 PMC4022288

[61] Briggs AD, Mytton OT, Kehlbacher A, Tiffin R, Rayner M, Scarborough P. Overall and income specific effect on prevalence of overweight and obesity of 20% sugar sweetened drink tax in UK: econometric and comparative risk assessment modelling study. BMJ. 2013;347:f6189 10.1136/bmj.f6189 24179043 PMC3814405

[62] World Health Organization Regional Office for Europe. Using price policies to promote healthier diets Copenhagen, Denmark: WHO Regional Office for Europe 2014 [cited 2015 May 5]. http://www.euro.who.int/__data/assets/pdf_file/0008/273662/Using-price-policies-to-promote-healthier-diets.pdf.

